# The impact of personal motivation on perceived effort and performance of pro-environmental behaviors

**DOI:** 10.3389/fpsyg.2022.977471

**Published:** 2022-10-11

**Authors:** Lieke Dreijerink, Michel Handgraaf, Gerrit Antonides

**Affiliations:** ^1^Energy Transition Studies, TNO, Amsterdam, Netherlands; ^2^Urban Economics Chairgroup, Wageningen University and Research, Wageningen, Netherlands

**Keywords:** motivation, perceived effort, difficulty, sustainable lifestyle, pro-environmental behaviors

## Abstract

In order to minimize climate change it is important that people take up a sustainable lifestyle. Sustainable lifestyles call for pro-environmental behaviors (PEBs) in several domains, such as in-home energy use, mobility, and consumption of food and goods. However, studies show that people often do not consistently behave pro-environmentally in all domains. In this study we investigated how a combination of personal motivation, and the difficulty and the perceived effort of a PEB, predicts the performance of PEBs in various domains, using a survey (*n* = 1,536). By means of Rasch analysis we identified the difficulty of 17 PEBs and estimated respondents’ pro-environmental motivations. In addition, we investigated if performance of certain PEBs increased the probability of performing other PEBs. This way we could identify for each level of motivation which behaviors respondents were (probably) performing and which behaviors they did not yet perform, but would be least effortful new behaviors. Furthermore, using a non-recursive structural equation model we investigated the relations between perceived effort, PEB performance, motivation, underlying traits, and demographics. Results showed a feedback loop between motivation and perceived effort: when respondents were motivated they perceived behaviors as less effortful and also lower perception of effort was related to higher motivation. Our results imply that people mainly perform PEBs that fit their level of pro-environmental motivation and that they are inclined to do the things of which they can justify the effort they need to invest. This amount of effort seems quite similar for people: no one wants to invest too much effort, but people highly differ in how effortful they assess different behaviors. Our study thus indicates that rationalizations play a key role. Encouraging people to embrace more sustainable lifestyles may involve step-by-step increases in PEB performance. We propose that people should be encouraged to perform behaviors that are closest to their current motivation level in order for them to progress from performing easy to more difficult PEBs.

## Introduction

Minimizing climate change requires transitions in people’s lifestyles (e.g., De Coninck et al., 2018; UNEP, 2020; IPCC, 2022). A sustainable lifestyle is characterized by patterns of behavior and habits embedded in society and facilitated by institutions, norms and infrastructures that frame individual sustainable choices (UNEP, 2016). Sustainable lifestyles require pro-environmental behaviors (PEBs) in various domains such as housing, food, mobility, leisure, and clothing. Next to consumption behaviors, behaviors in the citizenship domain that may affect institutions, norms and infrastructures, including voting or participating in social movements, are important to facilitate sustainable lifestyles (e.g., Stern, 2000; Nielsen et al., 2021). However, people do not consistently behave pro-environmentally across different domains. For instance, people may recycle their waste but at the same time make environmentally-unfriendly mobility choices (Steg and Vlek, 2009); and saving energy at home does not imply that people save energy while on holidays (Barr et al., 2011). In this study we explore these seemingly conflicting choices. We focus on the roles of motivation and of effort related to performing PEBs in various domains, as we expect that when PEBs are perceived as more difficult, people will need to put in more effort and therefore need to be more motivated to act pro-environmentally.

### Motivation and effort

Motivation is a (psychological) force that drives behavior and that consists of a direction (e.g., a goal) and intensity or amplitude with which this direction is pursued (i.e., effort) (Inzlicht et al., 2018).^1^ Although not all behaviors are goal-directed, all behaviors do require the investment of more or less effort aimed at overcoming financial, physical, cognitive and temporal barriers (e.g., Attari et al., 2011). A behavior that is easier to perform and thus requires less effort is more likely to be adopted, and vice versa: when behaviors are more difficult and require more effort people are less likely to perform them (Attari et al., 2011; Urban and Ščasný, 2016). In addition, people appear to be willing to exert effort up to a limit (Brehm and Self, 1989; Richter et al., 2016), but which factors and mechanisms underlie the investment of resources (i.e., effort) to carry out behavior is still one of the main questions of motivation science (Richter et al., 2016).

The concept of effort has been studied across various fields, but this has not led to one overall definition. Steele (2020) distinguishes between actual effort (i.e., objective effort) and the perception of that effort (i.e., subjective effort), with perceived effort building on actual effort. In psychological studies the focus lies mainly on perceived effort, and effort has been defined as the increase (“intensification”) of either mental or physical activity to meet some goal (Inzlicht et al., 2018). Effort thus refers to the intensity of behavior, but the goal is left unspecified. In this sense effort differs from motivation in that the latter is focused on a goal. It is generally assumed that effort is costly and that people avoid it to spare their resources; i.e., the principle of least effort (Zipf, 1949).^2^ In psychological studies, effort is often studied in the context of task performance.

The perceived difficulty of a task is a key concept in these psychological effort studies. Perceived difficulty enables individuals to avoid wasting effort as it provides information about the resources required for task success (Richter et al., 2016). In other words, when people know the difficulty of a task they can estimate how much effort they have to invest to complete the task. Difficulty of a task is thus seen as a property of the task itself. Although effort typically tracks difficulty (with people working harder when an action is more difficult) this relationship breaks down when incentives are too low or when an action is too difficult (Inzlicht et al., 2018). In that case, people give up on performing a task. Motivational Intensity Theory (MIT) describes that, as long as someone is able to perform the required behavior, the upper effort limit is determined by “potential motivation”; that is, the maximum amount of effort that is justified for task success (Brehm and Self, 1989). When people know that success is possible and benefits are large enough to justify the effort they need to invest, they remain motivated to act. MIT predicts that effort rises proportionally to subjective task difficulty as long as success is possible and necessary effort is justified. When a task is moderately difficult, the levels of required effort and potential motivation are much lower compared to a highly difficulty task. When the amount of effort required exceeds potential motivation, effort reached its peak and falls to zero: people stop performing the task.

### Pro-environmental motivation

We expect that MIT’s assumptions can also be applied to pro-environmental motivation and behaviors. Pro-environmental motivations are often described as moral motivations to do the “right” thing (e.g., Bolderdijk et al., 2013; Van der Linden, 2015). When people are more committed to reaching the goal of lowering one’s environmental impact or being environmentally friendly, their pro-environmental motivation is stronger. Although people can also perform PEBs because of other motivations, such as health or money saving, in this study we solely focus on the general pro-environmental motivation and goals. For this reason, we include multiple PEBs in our study. Similar to MIT, performance of a PEB could depend on the difficulty of the behavior and on a person’s potential motivation. Moreover, acting pro-environmentally requires people to be motivated to reach a certain goal—that is, lower one’s environmental impact or be environmentally friendly. In case of PEB, potential motivation stands for a person’s maximally justified effort that is needed to reach their goal of reducing one’s environmental impact. However, we suspect that, in line with a previous qualitative study (Dreijerink et al., 2021), the perception of effort may differ among people. In this study we asked a small sample of participants to score and explain the effort they attributed to several PEBs. Results indicated that those who did not perform PEBs associated these behaviors with higher effort levels compared to those who performed the PEBs.

Pro-environmental motivations are determined by a multitude of factors including values, beliefs, attitudes, and emotions (RLI, 2014). Although these determinants of motivation are not the focus of this study, the notorious gaps that are found in the relationship between (determinants of) motivation and behavior are relevant. For instance, the gap between attitudes and behaviors shows that people often hold pro-environmental attitudes but do not act upon them (e.g., Glasman and Albarracín, 2006). The value–action gap is a similar concept that points to a gap between values and behavior (Barr, 2006). A number of factors can reduce the gaps between motivation and behavior (e.g., Kollmuss and Agyeman, 2002). For example, Kaiser et al. (2021) described that the gap might stem from ignoring the fact that behavior typically involves costs, including personal resources. They found that attitudes must be strong enough to compensate for the costs of a behavior before the behavior has a reasonable chance of becoming manifest. This is similar to MIT’s presumption that people need to have a certain level of potential motivation to perform a behavior.

### Research goals

In order for a lifestyle to be sustainable people should not only perform easy PEBs, but also more difficult ones. In the current study we investigate how a combination of behavioral difficulty, effort, and motivation predicts the performance of PEBs in various domains. In doing so, we focus on differences between people. Firstly, we explore the levels of motivation and effort that are needed to perform individual PEBs. We want to understand where the limits of potential motivation lie and how those limits differ between people. More insight into people’s limits may provide clues how to encourage people to take up more difficult PEBs. Secondly, we investigate on an overall level how difficulty, effort, and motivation are related. In line with MIT we expect that for more difficult PEBs the maximally justifiable motivation—and thus required effort—is higher than for easier PEBs. As a result a smaller proportion of people will perform the PEB. In addition, we suspect that the perception of effort may differ among people: those who do not perform PEBs may associate these behaviors with higher effort levels compared to those who perform the PEBs. Furthermore, we explore the role of a number of determinants of motivation and demographics.

## Materials and methods

### Participants

This study was an addition to a study on social support for climate policy. The latter study used a sample from the I&O Research panel that was representative for Dutch society. In advance, we calculated by means of G*Power^3^ that our sample size was sufficient to detect small effects (*f* = 0.10), given 5% significance and 80% power. Participants were recruited at the end of November 2019. 1,536 People participated, including 54% males and 46% females. Their education level varied from 24% lower education (primary education up to and including incomplete secondary education), 35% medium education (secondary education, vocational education, up to and including first year higher vocational education), to 41% higher education (higher vocational education up to and including university degree). Age varied from 14% in the 18–39 year bracket, 39% in the 40–64 year bracket, and 47% were 65 or older.

### Materials and procedure

As this study was added to an online questionnaire on support for climate policy, parts of the questionnaire were unrelated to this study and are therefore not described. The relevant part of the questionnaire is included in the Appendix.

#### Personal values

We added one question on personal values to explore their role as a determinant of motivation. To assess personal values, respondents rated 16 items from Schwartz (1992) universal values scale adapted by Stern et al. (1999) as “guiding principles in their life” on a 9-point scale ranging from −1 (*opposed to my principles*), 0 (*not important*) to 7 (*extremely important*). We included three items for hedonic values (e.g., “Pleasure: gratification of desires”), five items for egoistic values (e.g., “Social power: control over others, dominance”), four items for altruistic values (e.g., “Equality: equal opportunity for all”) and four items for biospheric values (e.g., “Respecting the earth: harmony with other species”). Principal Component Analysis (PCA) showed that each of the items defined as altruistic, biospheric, egoistic, and hedonic did indeed load highest on the corresponding component. One exception was the altruistic item “A peaceful world” which loaded slightly higher on the biospheric component than on the altruistic component (0.51 versus 0.44). Since the difference was small, we decided to keep this item in the original group of altruistic values. The internal consistencies of the scales appeared good for all value groups: altruistic (Cronbach’s *α* = 0.69), biospheric (*α* = 0.85), egoistic (*α* = 0.79) and hedonic (*α* = 0.74). We therefore computed the mean score for each value group.

#### Concern about climate change

We added an item on concern about climate change to explore the role of this emotion as a determinant of motivation. To measure concern we used an item from research panel I&O Research (2020): “To what extent are you concerned about greenhouse gas emissions (including CO_2_), climate change and its effects on the environment?” Concern was measured on a 5-point scale ranging from 1 (*very much concerned*) to 5 (*not at all concerned*). In addition, respondents could indicate they did not know (these responses were excluded from the analysis). The scale was reversed in the analysis.

#### Performance of PEBs

Performance of PEBs was measured using items inspired by the general ecological behavior (GEB) scale items (Arnold et al., 2018). We included items from specific (consumption) domains, namely curtailing in-home energy use, efficient in-home energy use, mobility, food, buying goods, and green citizenship (Table 1). Furthermore, we added variation with regard to the environmental impact of behaviors: some having a low estimated impact (low carbon emission) versus others having higher impacts (higher carbon emission). Impact estimations were based on the Dutch website of Milieu Centraal (2019) that provides thorough information on environmental impacts based on lifecycle assessments. The goal of these emission estimations was to add variation in the selection of PEBs and not to quantify the exact impact of each behavior. To limit the questionnaire length we made a selection of 13 items from the 74 GEB items. Some items were adjusted to the Dutch situation. For example, prior interviews (see Dreijerink et al., 2021) showed that riding a bicycle or taking public transportation to go to work or school were perceived as very different and should therefore not be combined into one item (we have included three mobility items, i.e., items 7, 8, and 9 in Table 1). In addition, items were shortened for clarity. Finally, we added four items to have a sufficient number of items per domain (items 2, 14, 16, 17 in Table 1).

**Table 1:** 17 PEBs per domain and with an estimated carbon emission impact.

PEB	Domain	Est. carbon impact
1. Buy solar panels	Energy in home - efficiency	High
2. Buy a heat pump	Energy in home - efficiency	High
3. Insulate the house to keep it warm	Energy in home - efficiency	High
4. Put on a sweater in the house when it’s cold	Energy in home - curtailment	Low
5. Switch off lights and heating when you leave	Energy in home - curtailment	Low
6. Take short showers (maximum 5 min)	Energy in home - curtailment	Low
7. Use a bike for short distances (5 to 10 km)	Mobility	High
8. Use public transport for medium distances (30 to 60 km)	Mobility	High
9. Not go on a holiday by airplane	Mobility	High
10. Only buy fruit and vegetables grown in the Netherlands	Food	Low
11. Be a vegetarian (not eating meat or fish)	Food	High
12. Throw empty glass jars and bottles in bottle bank	Food	Low
13. Read about climate and environment	Green citizenship	Low
14. Vote for a political party committed to climate/ environ.	Green citizenship	High
15. Only buy products from eco companies	Goods	High
16. Buy second-hand items	Goods	High
17. Repair things and clothing that break down	Goods	High

Reversed in analyses.

Respondents were asked how often they performed the behaviors, on a 5-point scale ranging from 1 (*never*), 2 (*seldom*), 3 (*occasionally*), 4 (*often*) to 5 (*always*). In addition, respondents could indicate they did not know (these responses were excluded from the analysis). For four items, including having solar panels installed, having a heat pump installed, having their home insulated, or being a vegetarian, respondents were asked to indicate whether or not they performed this behavior, or that it was not applicable to them.

#### Perceived effort of PEBs

Perceived effort of each of the 17 PEBs was measured using a ten-point scale, from 1 (*very much effort*) to 10 (*very little effort*). The scale was reversed during analysis.

#### Motivation and difficulty

In order to test MIT, we measured the difficulty of the PEBs, potential motivation and perceived effort. Difficulty and motivation were determined by means of Rasch analysis. According to Campbell’s Paradigm, developed by Kaiser et al. (2010), one’s motivation to act pro-environmentally becomes apparent through the behaviors one actually performs.^4^ Campbell’s paradigm is implemented by means of a Rasch model, that specifies that a person’s odds of engaging in a behavior (*p*) versus not engaging in that behavior (*1-p*) are a function of their environmental motivation (*θ*) and the costs or difficulty (*δ*) of the specific behavior (Equation [1]); with *k* indicating a person and *i* indicating a PEB. The Rasch equation implies that when *θ_k_* equals *δ_i_* the probability that behavior *i* is performed by person *k* equals the probability that *i* is not performed. When *θ_k_* is larger than *δ_i_*, the probability of person *k* performing the behavior *i* increases. In other words, the stronger one’s motivation relative to the difficulty of a behavior item, the higher the probability that one performs that behavior.


(1) $$\mathrm{In}\left(\frac{p_{ki}}{1-p_{ki}}\right)=\theta_{k}-\delta_{i}$$

Rasch models have been used to predict energy-related and other pro-environmental behaviors (e.g., Kaiser, 1998; Kaiser and Wilson, 2000; Starke et al., 2020; Kaiser et al., 2021). In Rasch analysis behavioral probabilities are calculated by means of the (frequency of) performance of PEBs. Rasch analysis provides two outcomes: a rank order of behaviors according to their so-called behavioral costs or execution difficulty (*δ)*, and a rank order of individuals according to their pro-environmental motivation (*θ*). A strong advantage of the Rasch model is that it uses data on actual behavior performance to reveal one’s underlying motivation (*θ)*, as opposed to measuring motivation by means of a survey question that might be more subject to biases. In the current study we used Rasch analysis to determine the difficulty of the PEBs by means of *δ* and the motivation driving behavior by means of *θ*.

## Results

Results are reported in three sections. In the first section, results from the Rasch model to assess difficulty and environmental motivation are described. The second section includes an overview of the level of personal motivation that is needed for each of the 17 PEBs. We explore if we can identify PEBs that respondents did not perform (frequently) but that would fit one’s motivation. In the third section the relationship between PEB performance, environmental motivation, difficulty, and perceived effort is described.

### Rasch model

We constructed a unidimensional dichotomous Rasch model using the TAM package for R (version 4.02). For 4 PEBs performance was measured by means of a *yes/no* (or N/A) statement, while for the remaining 13 PEBs performance was measured on a 5-point scale (*never* to *always*). Since the practice of dichotomization in Rasch analysis is well-established and well-justified (Kaiser and Lange, 2021), we recoded responses to the 17 polytomous items to either yes (i.e., *always, often*) or no (i.e., *occasionally, seldom, never*). N/A answers on the four dichotomous items were excluded from the analyses. As a first step we investigated the fit of the items. As a rule of thumb, Linacre (2002) described a mean square (MSQ) fit value of 0.6 as a lower limit and 1.4 as an upper limit for item fit. All 17 PEB items were within these limits. Next, we investigated person separation reliability which indicates if a set of items is sensitive enough to distinguish between different individual performance levels.^5^ It is measured by means of weighted likelihood ability estimates (WLE). For the dichotomous model it turned out that the set of items was able to make a distinction between two groups of either low or high motivation (*WLE* = 0.59).

**Figure 1:**
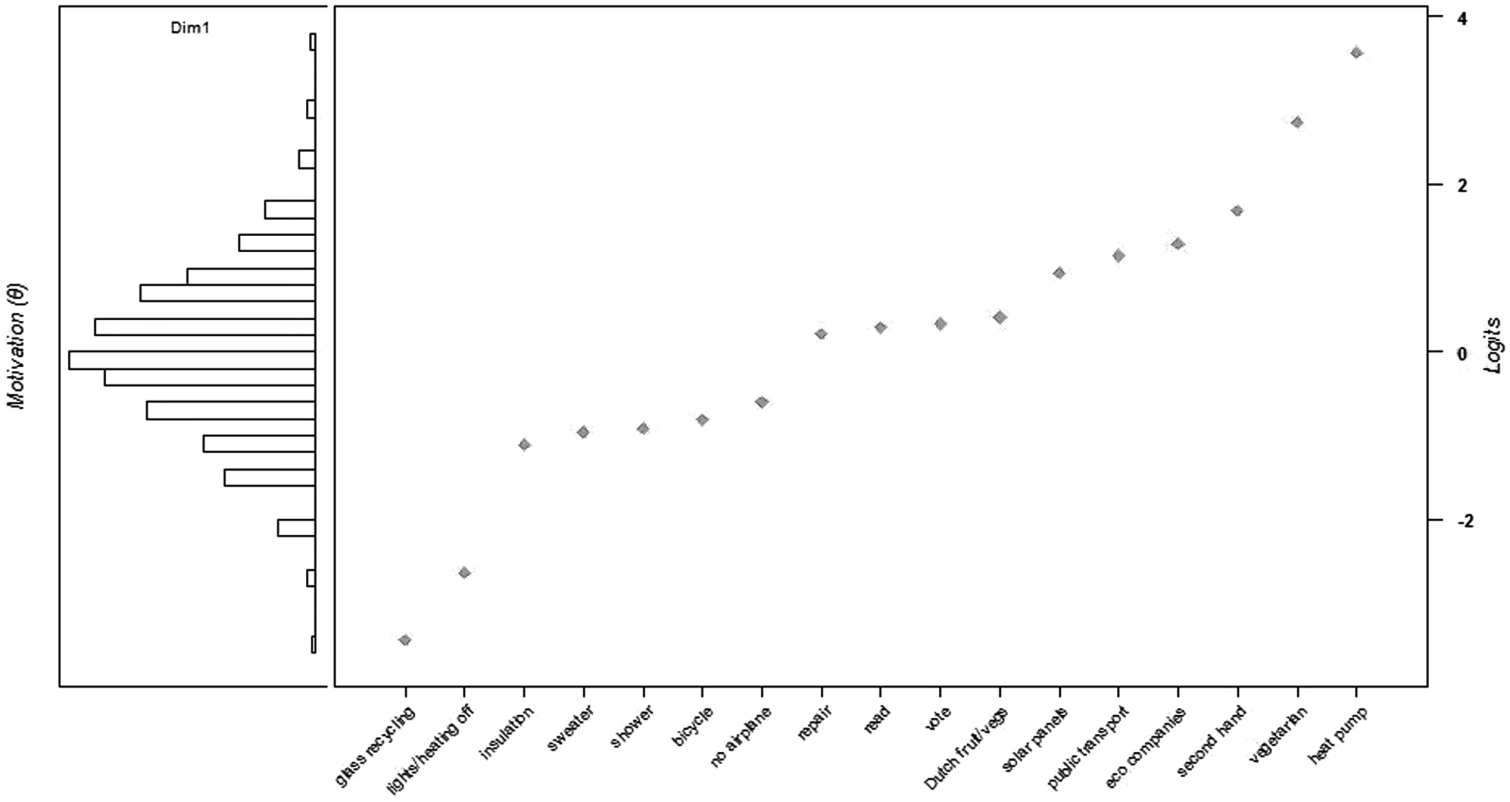
Wright map including person motivation (*θ*) and item difficulty (*δ*) of the 17 PEBs.

As described, the Rasch model has two outcomes: a rank order of behaviors according to their difficulty (*δ)*, and a rank order of individuals according to their motivation (*θ*). Figure 1 displays both outcomes in a so-called Wright Map or item-person map. The item-side on the right shows the difficulty of the PEBs: glass recycling appeared to be the easiest and installing a heat pump was the most difficult PEB. Furthermore, the person-side on the left shows that personal motivation scores (*θ*) ranged from −3.48 to 3.68 (*M* = 0.00, *SD* = 0.97).^6^ A lower negative *θ* reflects a weaker pro-environmental motivation, while a higher positive *θ* reflects a stronger motivation. Moreover, the Wright Map shows the lowest level of motivation (*θ*) at which a certain PEB is performed. For instance, with a motivation (*θ*) of zero a respondent performed about half of the PEBs (from glass recycling up to not travelling by airplane). Repairing things and clothing was a PEB that about half of the respondents performed.

### Behaviors by level of motivation and perceived effort

As Figure 1 shows, motivation (*θ*) levels are positively correlated with the performance of certain PEBs and the difficulty of the PEBs (with some deviations from a linear relationship). For example, respondents with the lowest motivation levels only recycled their glass and turned off lights and heating. For this group, the nearest, least difficult next PEB would be to insulate their home. Moreover, the Wright map shows that some PEBs are close to each other in terms of difficulty (*δ)*; for instance, difficulties of repairing, reading, green voting, and buying Dutch fruits and vegetables are all in between 0.2 and 0.45. Therefore, it would be likely that respondents with the corresponding motivation level on the person-side would perform all of these PEBs. Or, in case they did not perform all behaviors, it would be likely that the not-performed PEB would fit their motivation and would be the least difficult, new PEB. In addition to the clusters of PEBs with similar difficulties, the Wright map displays leaps between PEBs, implying that the subsequent behavior would be a lot harder to perform; for instance, from not going on holiday by airplane to repairing, or from buying Dutch fruits and vegetables to installing solar panels. Using the Wright map we can therefore identify the easiest, new PEBs for each motivation level.

In addition, with regard to levels of perceived effort we found that when respondents performed the PEBs (*always, often*), they on average assessed the effort of the 17 PEBs at 2.4, with a maximum perceived effort score of 3.8 for buying from eco companies. On the other hand, non-performers (*occasionally, seldom, never*) assessed the effort of all 17 PEBs on average at 6.4, with the minimum score of 4.7 for putting on a sweater. The level of effort a respondent attributes to a behavior may therefore provide an indication of how likely someone is to perform the behavior.

Furthermore, we investigated if the performance of each PEB on the slope of the Wright map could serve as some kind of stepping stone or gateway for the next PEB to occur. For each PEB we calculated conditional probabilities; that is the probability of a behavior (PEB2) occurring (yes/no) given that a previous behavior (PEB1) occurred (yes/no). We compared conditional probabilities with unconditional probabilities of PEB2 occurrence and found that for 12 PEBs the probability of PEB performance was higher when respondents had performed a previous behavior (Table 2). The largest conditional probabilities appeared between reading and voting (16%), public transport and buying from eco companies (10%), and buying from eco companies and buying second hand (10%). Since these three steps were not part of the leaps in Figure 1 we described, this result seems another indication that the occurrence of these combinations of PEBs might be more likely than other combinations of PEBs. Strikingly, when respondents had installed solar panels the probability of using public transport was lower compared to unconditional probability. A possible explanation could be that the motivation to install solar panels is different compared to why people perform other PEBs, such as using public transport. In addition, solar panels may be installed more often by people with higher incomes, who may be less inclined to use public transport. Finally, installing solar panels may provide a license to refrain from additional PEBs.

**Table 2:** Unconditional and conditional probabilities of PEB performance.

Step		P(PEB2 = Yes)	P(PEB2 = Yes|PEB1 = Yes)	Difference
PEB1	PEB2			
Recycle glass	Lights and heating off	0.92	0.92	0
Lights and heating off	Insulate	0.73	0.73	0
Insulate	Put on a sweater	0.70	0.70	0
Put on a sweater	Take short showers	0.69	0.73	0.04
Take short showers	Use a bicycle	0.67	0.72	0.05
Use a bicycle	No holiday by airplane	0.63	0.64	0.01
No holiday by airplane	Repair	0.45	0.50	0.05
Repair	Read	0.44	0.49	0.05
Read	Vote green	0.43	0.59	0.16
Vote green	Buy Dutch fruit and vegs	0.41	0.46	0.05
Buy Dutch fruit and vegs	Install solar panels	0.30	0.34	0.04
Install solar panels	Use public transport	0.26	0.21	−0.05
Use public transport	Buy from eco companies	0.24	0.34	0.10
Buy from eco companies	Buy second hand	0.18	0.28	0.10
Buy second hand	Be a vegetarian	0.08	0.13	0.05
Be a vegetarian	Install a heat pump	0.04	0.04	0

### Relation between motivation, perceived effort and PEB

Finally, we investigated the relationship between the (perceived) difficulty of a PEB, motivation, and PEB performance. Item difficulty (*δ*), as measured in the Rasch model, correlated strongly with the average performance of each separate PEB (*r* (16) = −0.825, *p* = 0.000), indicating that difficult PEBs were performed less frequently. In addition, we asked respondents about the perceived effort of each PEB. Average perceived effort of the 17 PEBs appeared to correlate very strongly (*r* (16) = 0.96, *p* = 0.000) with item difficulty (*δ*). We decided to use perceived effort as the indicator of difficulty as it provided variation between respondents. Motivation was measured using estimated *θs* from the Rasch model. In addition, we were interested in exploring the role of personal values, concern about climate change and demographics within the relationship between perceived effort, motivation, and PEB performance.

**Figure 2:**
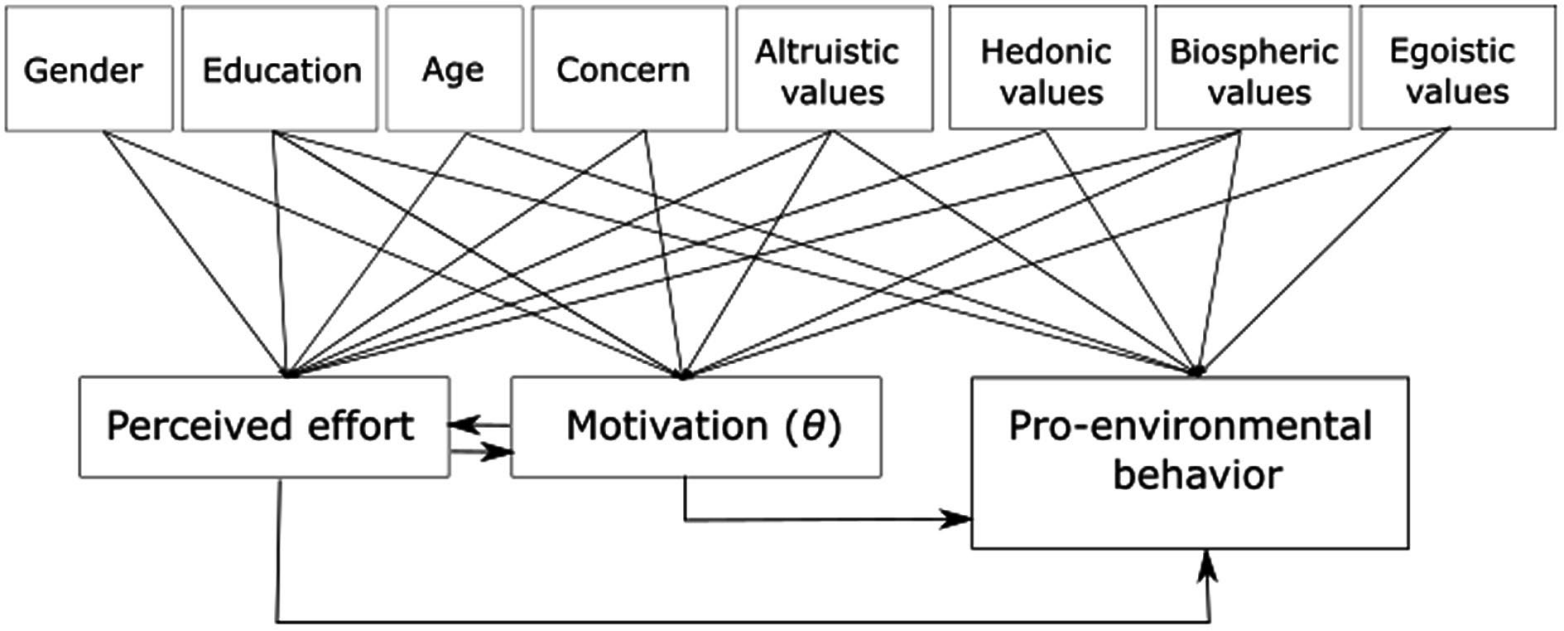
Overview of non-recursive model 4, with missing arrows from predictors (*x*) showing a relation that was restricted to 0.

We used LISREL (version 11.4.2) to estimate and explore several models, including both recursive and non-recursive models. A recursive model is a type of structural equation model (SEM) that is characterized by effects that go into one direction, as opposed to a non-recursive model that includes reciprocal effects or feedback loops. In three recursive models and one non-recursive model we included PEB, motivation, and perceived effort as dependent variables (*y*), and personal values, concern about climate change and demographics as predictors (*x*). Based on theoretical insights, we expected the non-recursive model to be the best. First, as described in the introduction, MIT states that motivation is affected by perceived effort of a behavior, since people remain motivated to act depending on justifications of the effort they need to invest. Second, we described that motivation may affect the perception of effort, since people who perform PEBs and are therefore more motivated assess behaviors as less effortful. Both relations are included in the non-recursive model. In addition, we tested three underlying, simpler models to explore if any of them would be better than the non-recursive model. These four models included all possible combinations between motivation and perceived effort in relation to PEB performance: in model 1 PEB was predicted by motivation and perceived effort, and predictors (*x*) were added to explain PEB, motivation, and perceived effort. Model 2 was similar to model 1 but with perceived effort predicting motivation; model 3 was similar to model 1 but with motivation predicting effort; and model 4 was similar to model 1 but with a feedback loop between motivation and perceived effort. In order to identify the feedback loop, we restricted some relationships between *x* variables and *y* variables to zero (Figure 2). In addition, we explored if there might be one or two underlying factors that could explain the relations between all variables. We therefore tested three models in which we combined the observed variables into latent traits. These three models included all possible combinations with underlying factors. Recursive model 5 and non-recursive model 6 included two latent variables, namely *η_1_* based on the observed variables PEB and motivation, and *η_2_* that was equal to perceived effort. In model 5 we included a direct effect of *η_2_* on *η_1_.* In model 6 we included a reciprocal relation between *η_1_* and *η_2_*. Finally, in model 7 the latent variable *η* was based on the observed variables PEB, motivation, and perceived effort. In the three models variables (*x*) were included as predictors of the latent variables. In non-recursive model 6 we applied restrictions to some relationships between *x* variables and *y* variables, similar to model 4.

Since the different models were not nested in general (i.e., each model typically could not be considered a restricted form of another model) we used AIC (Akaike Information Criterion; Akaike, 1974) and BIC (Bayesian Information Criterion; Schwarz, 1978) values that are suitable for comparing the quality of non-nested models (Henson et al., 2007). As lower AIC and BIC values indicate a better fit, non-recursive model 4 proved to be the best, while models 5 and 6 came in second and third (see Table 3). In addition, modification indices and expected parameter changes provided insight into whether models could be improved by removing restrictions between variables. We found that model 1 would improve by adding relations between motivation and perceived effort, as we did in models 2, 3 and 4. Modification indices showed no additional improvements for models 2, 3, and 4. Models 5 and 6 could both be improved by relating *η_2_* (perceived effort) to PEB and motivation and by relating the measurement errors of the predictors *x* to the measurement errors of PEB and motivation, but this would go against our idea of the existence of two latent variables. Model 7 could be improved by relating measurement errors of the predictors *x* to the measurement errors of PEB, motivation and perceived effort, but this would go against the idea of one latent variable. Finally, squared multiple correlations provided an indication of the proportion of variance in the variables *y* accounted for by the variables on the structural equations. As displayed in Table 3, model 4 appeared to explain most variance of PEB (34%), motivation (30%) and perceived effort (31%) when compared to models 2 and 3. In short, model 4 turned out to be of best quality. Since model 4 was saturated, the goodness of fit was perfect, that is the empirical correlation matrix did not differ significantly from the fitted (modeled) covariance matrix (Ganzeboom and Nikoloski, 2012).

**Table 3:** AIC, BIC, squared multiple correlations, df, AGFI and RMSEA values for the seven models.

Model	AIC	BIC	Squared multiple correlations for reduced form					df	AGFI	RMSEA
			PEB	Motivation	Perceived effort	Eta1	Eta2			
1	9336.833	9683.649	0.37	0.27	0.15			1	0.943	0.634
2	8723.836	9075.988	0.27	0.22	0.15			0	1.00	0.000
3	8723.836	9075.988	0.32	0.27	0.30			0	1.00	0.000
4	8651.836	8811.905	0.34	0.30	0.31			0	1.00	0.000
5	8704.256	8821.640				0.39	0.31	8	0.935	0.069
6	8706.256	8828.976				0.39	0.31	7	0.926	0.076
7	9751.063	9820.426				0.31		17	0.587	0.194

Model 4 showed a significant feedback loop between motivation and perceived effort. Motivation appeared to have a highly significant negative impact on perceived effort (*β* = −1.39, *t* = 21.03, *p* = 0.000) and perceived effort appeared to have a (less significant) negative impact on motivation (*β* = −1.62, *t* = −4.69, *p* = 0.000). In addition, motivation appeared to have a positive impact on PEB performance (*β* = 0.84, *t* = 2.93, *p* = 0.003), while the relation between perceived effort and PEB was not significant (*β* = 0.24, *t* = 1.18, *p* = 0.237). In addition to direct effects, LISREL provides insight into the indirect effects and total effects in a model. We were especially interested in the total effects (i.e., the sum of the direct and indirect effects) of predictors (*x*) on the dependent variables. As shown in Table 4, stronger biospheric values, a higher concern about climate change, being female, and having a higher education were related to more frequent PEBs and a stronger motivation. With age, respondents appeared to be more motivated. On the other hand, holding stronger hedonic values was a negative predictor of PEB and of motivation. Furthermore, we found that higher perceived effort was associated with lower biospheric and altruistic values, lower concern about climate change, being male, a lower education level and age, and stronger hedonic values.

**Table 4:** Total effects (coefficients and *t*-values) of predictors (*x*) on PEB, motivation and perceived effort of model 4.

	PEB		Motivation		Perceived effort	
	*β*	*t*	*β*	*t*	*β*	*t*
Biospheric values	**0.09**	9.51	**0.19**	9.48	**−0.26**	−9.66
Egoistic values	0.00	−0.64	0.00	0.25	0.00	−0.25
Hedonic values	**−0.05**	−6.35	−**0.15**	−8.04	**0.09**	3.67
Altruistic values	0.01	0.84	0.00	−0.11	**−0.08**	−2.13
Concern about climate change	**0.11**	12.14	**0.22**	11.35	**−0.3**	−11.04
Gender	**0.13**	7.05	**0.21**	5.36	**−0.21**	−3.68
Education	**0.05**	3.87	**0.11**	4.55	**−0.21**	−5.56
Age	0.02	1.25	**0.15**	4.82	**−0.21**	−4.95

Significant coefficients are shown in bold face.

## Discussion

In this study we investigate how a combination of behavioral difficulty, perceived effort and motivation predicts the performance of PEBs in different domains. In doing so, we focus on differences between people. First, in our overview of the levels of motivation that are needed for people to perform PEBs we show that specific levels of personal motivation are associated with the performance of specific PEBs. We find that the performance of certain PEBs seems to increase the probability of performing other PEBs. It appears that certain combinations of PEBs fit together; although effects are small. Since our study is correlational we cannot prove causality. However, if causal relations between behaviors would be the case this would imply behavioral spillover; that is, the performance of one PEB leading to another (e.g., Thøgersen, 1999). The literature on behavioral spillover indeed shows that for some combinations of PEBs (positive) spillover occurs, but in general effects are small and results are mixed (e.g., Maki et al., 2019; Geiger et al., 2021). However, in previous studies on behavioral spillover personal motivation levels were not included. We propose that when designing interventions (such as a campaign or an experiment) aimed at encouraging behaviors that are part of sustainable lifestyles, insights from our study could be taken into account. For example, a tailored intervention could include PEBs that are closest to a person’s level of motivation and their current behaviors. Starke et al. (2020) indeed showed that when energy-saving measures are more tailored to one’s motivation people perceive them as more adequate. In addition, it could be effective to encourage people to increase the frequency of PEBs that they already engage in occasionally. Follow-up experimental and field studies could investigate the effects of this approach and what role a personal motivation level plays.

Furthermore, we want to understand where the limits of potential motivation lie and how those limits differ for people. We find that if a PEB is perceived as too effortful people generally do not perform it. People thus indeed hold a limit with regard to how much effort they are willing to invest, as previous studies have described (Brehm and Self, 1989; Richter et al., 2016). In this line of thinking, the difficulty of a PEB is a property of the behavior (task) itself, consistent with MIT. Additionally, our study shows that behaviors can be ranked from easy to difficult and people are more inclined to perform the easy behaviors than the difficult ones. However, in line with our previous findings (Dreijerink et al., 2021), we also find that people who perform a PEB generally assess the behavior as less effortful, as opposed to people who do not perform the same behavior, who do consider it effortful. People indeed adjust their perception of the effort of a PEB. In this sense, “difficulty” does not seem to be a property of the behavior (task) itself, but is a result of motivation and whether a behavior has been performed. We find that motivation plays a key role as a predictor of both PEB performance and perceived effort: when people are motivated they are more inclined to behave pro-environmentally and perceive PEBs as less effortful. It might be that they downplay the effort level of PEBs compared to people who are less motivated, or that people who are less motivated exaggerate the effort of behaviors. Recalling MIT’s description of potential motivation as a person’s maximally justified effort that is needed to reach one’s goal, it appears that perception of effort might play a role within these justification processes. If a person’s goal is to lower their environmental impact, it might not fit one’s pro-environmental identity to “complain” about effort. Another explanation could be that if one would want to lead by example, downplaying the level of effort might inspire others to do the same thing. On the other hand, if a person’s environmental goal is less strong it might help to exaggerate the effort as a justification for not performing a PEB. Although we find that motivation indeed needs to compensate for effort in order for a behavior to become manifest, as Kaiser et al. (2021) described, this compensation mainly seems to occur within people’s perception of effort. The feedback loop between motivation and perceived effort shows a more complex process. In addition to motivation affecting the perception of effort, a lower perceived effort level of a PEB may motivate people to perform this particular PEB, although the latter effect is somewhat weaker than the former. It appears that perceived effort mainly affects behavior indirectly *via* motivation. Follow-up studies could investigate this reciprocal process and the accompanying rationalizations. For example, why the effect of motivation on perceived effort is stronger than the other way around, or what the processes of exaggerating and downplaying may entail. Our previous qualitative study (Dreijerink et al., 2021) showed that when people perceive behaviors as more effortful they increasingly seemed to use arguments to rationalize why performing the behavior is difficult or impossible. It may also be interesting to study rationalizations to perform behaviors that are perceived as easy, or if people may experience internal struggles between pro-environmental and environmentally-unfriendly rationalizations.

Based on our findings, motivation and (perceived) effort can be seen as levers that can encourage people to perform PEBs more frequently or to perform PEBs they had not performed before. In this sense, motivation turns out to be a more important lever than perceived effort because of the relatively strong relation running from motivation to perceived effort. Although this study did not include ways to increase motivation or to reduce (perceived) effort, results may be helpful. Motivation seems to be affected by several factors that are difficult to change, including personal values and demographics. However, concern about climate change is a factor that may change when the sense of urgency within society would be greater. At the moment, there is a generally felt concern about climate change but at the same time (high greenhouse gas emitting) societies and governments exude little urgency. An increased feeling of urgency could lead to higher levels of motivation and additionally to lower perceptions of effort. In addition, perception of effort could be lowered when in general the performance of PEBs will be less effortful and difficult; for example, PEBs could be made less expensive, less time consuming or less demanding. In other words, people’s agency or ability to act would improve. This might in particular be helpful for specific groups of people that are at the moment least inclined to perform PEBs and who justify their inaction by the high levels of needed effort. In sum, the often proposed combination of increasing urgency and improving agency will be a suitable approach to encourage sustainable lifestyles.

### Limitations

Our study has several limitations. Due to questionnaire length we could only include a limited number of PEBs. We made a selection of the most relevant PEBs for Dutch households, but a larger selection would have improved the study. For example, we were interested in making a distinction between behaviors in different domains, and wanted to examine if there would be differences in perceived effort and motivation per domain. In case of adding more PEBs, we could have developed a multidimensional Rasch model to explore the dimensionality of motivation. In unidimensional Rasch models it is assumed that the difference between two subjects in responding to a set of items depends on a single latent trait (Bartolucci, 2007), while in a multidimensional model multiple latent traits affect subjects’ responses (Katz et al., 2021). In our study the number of items was too small for such an analysis and we therefore used a general motivation measure (*θ*). In general, the distinguishing power of the scale between people with different levels of motivation would improve if more items were added.

Moreover, our study focused on the interrelations between motivation, perceived effort and PEB performance. Although we did control for personal values and concern about climate change, our study was not about the determinants of motivation, such as values, attitudes, beliefs and emotions. What combination of factors exactly defines motivation is, however, an interesting issue that calls for further research. In relation, our focus on pro-environmental motivation excluded other types of motivations, such as health or financial reasons that may (co-)drive PEB performance. To understand why people perform behaviors, or to understand what motivates different people to perform different behaviors at different times, Kaiser (2021) describes an approach in which all possible motivations are considered and included in as many models as there are personal goals. Such an approach, although complex, could provide important insights into how different combinations of motivations may affect PEB performance.

Finally, MIT is often tested by means of effort tasks and has not previously been applied to self-reported PEBs. We see some differences between performing a task in a lab and performing a PEB in real life. For example, the description that people work harder when a task is more difficult (Inzlicht et al., 2018) does apply to a lab task but does not seem to apply to PEB. In addition, the difficulty of a PEB seems to be surrounded by more subjectivity than the difficulty of, for example, a memory or a letter-scanning task. But although our study is less relevant for supporting MIT we think our results contribute to insight into PEB performance.

### Conclusion

In the introduction we described that people do not consistently behave pro-environmentally across domains, as they recycle their waste but also make environmentally-unfriendly mobility choices, or save energy at home but not while they are on holidays. We would now state that waste recycling and mobility choice, and energy use at home and at a holiday destination are on different difficulty levels and it is no surprise that not everyone conducts both types of behaviors as it does not fit everyone’s motivation. People appear to perform specific sets of PEBs depending on their motivation. For some this set of PEBs is limited while for others this set is more expansive. Although most people have pro-environmental intentions and thus have some sort of environmental motivation, this does not mean they are willing to (frequently) perform all kinds of PEBs. People are inclined to only do the things for which they can justify or rationalize the effort they need to invest. The amount of perceived effort seems quite similar for people: they do not want to invest too much effort, but they highly differ in how effortful they assess different behaviors. Our study indicates that rationalizations appear to play a key role. Encouraging people to embrace more sustainable lifestyles may involve step-by-step improvements in PEB performance. We propose that people should be encouraged to perform behaviors that are closest to their current motivation level and that can therefore be justified. This way people can progress from performing easy to more difficult PEBs.

## Data availability statement

Data that support the findings of this study are available through the Open Science Framework at: https://osf.io/fus3g/?view_only=eca90028006d444ca62e54e426cbaf04.

## Ethics statement

Ethical review and approval was not required for the study on human participants in accordance with the local legislation and institutional requirements. The participants provided their written informed consent to participate in this study.

## Author contributions

LD: conceptualization, writing–original draft, writing–review and editing, validation, formal analysis, investigation, data curation, and visualization. MH and GA: conceptualization, writing–original draft, writing–review and editing, validation, and investigation. All authors contributed to the article and approved the submitted version.

## Conflict of interest

LD was employed as a researcher at TNO at the time of data collection.

The remaining authors declare that the research was conducted in the absence of any commercial or financial relationships that could be construed as a potential conflict of interest.

## Publisher’s note

All claims expressed in this article are solely those of the authors and do not necessarily represent those of their affiliated organizations, or those of the publisher, the editors and the reviewers. Any product that may be evaluated in this article, or claim that may be made by its manufacturer, is not guaranteed or endorsed by the publisher.

## Footnotes

^1^When we describe motivation we mean intrinsic motivation (i.e., driven by internal forces), and not extrinsic motivation (i.e., driven externally, such as by means of a financial incentive).

^2^Effort can, however, also have positive value by itself, as people tend to also associate effort with reward and will sometimes select objects or activities because they require effort (e.g., Inzlicht et al., 2018; Gathen and Praxmarer-Carus, 2020).

^3^G*Power is freely available software from the Heinrich-Heine University Düsseldorf (Faul et al., 2009).

^4^In Campbell’s Paradigm and the literature on Rasch models it is convention to use the term attitude to refer to a person’s probability of performing the given set of PEBs, we prefer to view *θ* as an expression of motivation. We argue that *θ* is an expression of engagement with certain behaviors that goes beyond weighting beliefs about advantages and disadvantages of behaviors, as is the definition of attitudes (RLI, 2014).

^5^Rasch analysis is commonly used in the development of educational test instruments. For these types of tests it is important that the test items are able to distinguish if a student performs low or high, and thus would fail or pass the test.

^6^*θ* is assessed in logits, which represent the natural logarithm of the ratio of the number of pro-environmental and environmentally unfriendly self-reported behaviors (Kaiser et al., 2021).
