# Supplementary material for: The impact of personal motivation on perceived effort and performance of pro-environmental behaviors
Source: Front Psychol. 2022 Oct 11;13:977471. doi: 10.3389/fpsyg.2022.977471 (PMC9595114; doi:10.3389/fpsyg.2022.977471)
Supplement: Supplementary file 1 [file Data_Sheet_1.pdf]

Appendix. Questionnaire (translated from Dutch)

Q. How important are the following 16 values for you as a guide in your life?
Your scores can range from –1 to 7. The higher the number (0, 1, 2, 3, 4, 5, 6, 7) the more important the value. The score -1 means that the value goes against your principles. The score 0 means that the value is not important to you at all. Try to vary your judgments as much as possible, and only score the values that are extremely important to you a 7.

EQUALITY. Equal opportunities for everyone
RESPECT FOR THE EARTH. Living in harmony with other species
SOCIAL POWER. Control over others, dominance
FUN. Enjoyment, fulfilment of desires
UNITY WITH NATURE. Feeling connected to nature
A PEACEFUL WORLD. Freed from wars and conflict
WEALTH. Material assets, money
AUTHORITY. The right to lead and determine
SOCIAL JUSTICE. Recovering injustice, caring for the weak
ENJOY LIFE. Good food, sex, free time, etc.
ENVIRONMENTAL PROTECTION. Preservation of the environment and nature
INFLUENCE. Have an impact on people and events
USEFULNESS. Promote the well-being of others
AVOID ENVIRONMENTAL POLLUTION. Protect natural resources
ENJOYMENT. Doing as many fun things as possible.
AMBITIOUS. Work hard, pursue ambitions and goals

Q. To what extent are concerned about greenhouse gas emissions (including CO_2), climate change and its effects on the environment?
1. Highly concerned
2. Somewhat concerned
3. Neutral
4. No so much concerned
5. Not concerned at all
6. Don't know

Q. Can you indicate to what extent you perform the following 17 behavior?
Never / rarely / occasionally / often / always

I put on a sweater in the house when it is cold
I turn off lights and heating when I leave
I take short showers (maximum 5 minutes)
I'm going on a holiday by airplane
I use public transport for medium distances (from 30 to 60 km)
I use the bike for short distances (from 5 to 10 km)
I throw empty jars and bottles in the glass container
I only buy fruit and vegetables that are grown in the Netherlands
I read about the climate and environment
I vote for a political party that is committed to climate and environment
I buy products from sustainable companies
I buy second-hand stuff
I repair things and clothing that break

Yes / No / Not applicable
I have solar panels on my roof
I have a heat pump
I insulated my house to keep it warm
I am a vegetarian (I do not eat meat or fish)

Q. Some pro-environmental behaviors take more effort than others. If you look at the 17 examples of behavior, how much effort would it take you to perform the behavior?
Use the slider to assess every behavior; from it takes me very much trouble (score 1) to it takes me very little trouble (score 10).

Install solar panels on the roof
Purchase a heat pump
Insulate the house to keep it warm
Wear a sweater in the house when it is cold
Switch off the lights and heating when away
Take short showers (maximum 5 minutes)
Not go on holiday by airplane
Use public transport for medium distances (from 30 to 60 km)
Use the bike for short distances (from 5 to 10 km)
Throw empty jars and bottles into the glass container
Only buy fruit and vegetables grown in the Netherlands
Being vegetarian (do not eat meat or fish)
Read about the climate and environment
Voting for a political party that is committed to climate and environment
Only buy products from sustainable companies
Buy second-hand stuff
Repair items and clothing that are broken
